# Clinical efficacy of belimumab in central nervous system demyelinating syndromes with systemic lupus erythematosus: A case series

**DOI:** 10.1097/MD.0000000000034079

**Published:** 2023-06-23

**Authors:** Ya Liu, Qiuyu Fan, Chao Jia, Qibin Wan, Huiqin Yang

**Affiliations:** a Department of Rheumatology and Immunology, Traditional Chinese and Western Medicine Hospital of Wuhan, Tongji Medical College, Huazhong University of Science and Technology, Wuhan, China.

**Keywords:** Belimumab, systemic lupus erythematosus, central nervous system demyelinating syndromes

## Abstract

Systemic lupus erythematosus (SLE) is a complex autoimmune disease involving multiple systems. Central nervous system (CNS) demyelinating syndromes are one of the rare neurological manifestations of SLE, whose diagnosis, treatment, and prognosis are rarely reported. Belimumab, an anti-BAFF monoclonal antibody, has been approved by the FDA for the treatment of SLE. We aimed to assess the effects of belimumab on demyelinating syndromes in patients with SLE.

**Patient concerns:**

Six patients with demyelination in SLE who were managed at Traditional Chinese and Western Medicine Hospital of Wuhan from March 2021 to March 2023, who received belimumab ≥ 5 times, were enrolled. Ten age- and sex-matched SLE patients with noutneuropsychiatric systemic lupus erythematosus (NPSLE) and normal controls were recruited to analyze potential biomarkers.

**Diagnoses:**

All patients were diagnosed with SLE based on the 2012 Systemic Lupus International Collaborating Clinics (SLICC) SLE classification criteria or the 2019 EULAR/ACR classification criteria. All SLE patients with CNS demyelinating syndromes were diagnosed by rheumatologists, neurologists, and radiologists.

**Interventions:**

These patients were administered belimumab combined with standard treatment (glucocorticoids and/or antimalarials and/or immunosuppressants [cyclophosphamide, mycophenolate, methotrexate, etc.]).

**Outcomes:**

Six patients were included in the study (100% female, mean [range] age at first demyelinating episode 42.8 [24–66] years). The most common extra-CNS features in these patients were rash, arthritis, alopecia, leukopenia, and hypocomplementemia. After Belimumab treatment, 3 of 6 (50%) patients achieved complete remission with decreased prednisone, 2 improvements, and 1 relapsed with uterine surgery. Compared with the baseline, 3.5 months post belimumab treatment, the disease activity score SLEDAI (21.5–5.5, *P* < .001), C3 and C4 increased, and extra-CNS symptoms improved rapidly. Moreover, The expression of lupus susceptibility gene PBX1 in CD19+ B cells was lowest in demyelinating syndromes with lupus patients compared with healthy volunteers and lupus patients without demyelination, and its relative expression negatively correlated with SLE disease activity.

**Conclusion:**

Belimumab could be an effective and safe option for the treatment of SLE demyelination. In addition, PBX1 might be a potential biomarker for the clinical diagnosis of lupus in patients with demyelinating syndrome.

## 1. Introduction

Systemic lupus erythematosus (SLE) is a chronic autoimmune disease with an immune-mediated pathogenesis characterized by a wide range of clinical manifestations.^[1,2]^ Neuropsychiatric manifestations in SLE are present in approximately 12% to 95% of patients,^[3,4]^ and they may lead to a worse overall prognosis.^[5]^ Among the many neuropsychiatric syndromes that constitute neuropsychiatric systemic lupus erythematosus (NPSLE), demyelinating syndromes (SLE-DS), termed lupoid sclerosis,^[6]^ is acute or relapsing encephalomyelitis with evidence of discrete neurological lesions distributed in place and time. DS occurs after the establishment of SLE, but may be the first manifestation of the disease.^[7]^ In addition, little is known about the clinical characteristics, treatment, and prognosis of SLE-DS. Studies have shown that BAFF is commonly overexpressed in SLE and is strongly involved in its pathogenesis.^[8]^ Belimumab, an anti-BAFF monoclonal antibody, has been approved for the treatment of active autoantibody-positive SLE.^[9]^ Recently, Belimumab has been proven efficacious in a variety of SLE disease conditions, such as nephritis and other neuropsychiatric manifestations of SLE,^[10,11]^ while the effect of belimumab on demyelination with SLE remains unclear.

## 2. Methods

Six patients with central nervous system (CNS) demyelination with SLE in Traditional Chinese and Western Medicine Hospital of Wuhan from March 2021 to March 2023, who received belimumab ≥ 5 times. These patients were diagnosed with SLE based on the 2012 Systemic Lupus International Collaborating Clinics (SLICC) SLE classification criteria^[12]^ or the 2019 EULAR/ACR classification criteria.^[13]^ All patients were treated with belimumab (10 mg/kg at 2-week intervals for the first 3 doses and at 4-week intervals thereafter). Belimumab was diluted and injected over 1 hour. Blood pressure and ECG were monitored within the first 3.5 hours of administration to check for any reaction to the drug infusion. All SLE patients with CNS demyelinating syndromes not fulfilling the criteria for multiple sclerosis, neuromyelitis optica spectrum disorders, and neuromyelitis optica were evaluated by rheumatologists, neurologists, and radiologists. These patients were administered belimumab combined with standard treatment (glucocorticoids and/or antimalarials and/or immunosuppressants [cyclophosphamide, mycophenolate, methotrexate, etc.]). 10 age- and sex-matched SLE patients without NPSLE and normal controls were recruited to analyze the expression of PBX1 in peripheral blood B cells. Healthy donors had no history of autoimmune disease or any treatment with immunosuppressive agents.

This work was approved by the Research Ethics Committee of Traditional Chinese and Western Medicine Hospital of Wuhan, and informed consent was obtained from all participants.

## 3. Results

The study consisted of 6 female patients with a mean (range) age of 42.8 (24–66) years. The mean (range) duration of illness from the onset of SLE to administration of Belimumab was 1.2 years (1 months to 5 years). Short-term intravenous high-dose dexamethasone and/or methylprednisolone (MP) and immunosuppressants including Cyclophosphamide, Mycophenolate mofetil, Methotrexate and Hydroxychloroquine, and intravenous immunoglobulin (400 mg/kg/d for 5 days) therapy were administered before enrollment in the belimumab protocol. In addition, 2 patients with intractable disease did not respond to the combination treatment and thus received PE therapy as well. All 6 patients had multiple organ involvement, including the joint, skin, blood system, kidney, and neuropsychiatric system. CNS demyelinating syndromes were the initial manifestation of SLE in 3 patients. Regarding the Anatomical distribution of CNS lesions on MRI, 5 patients had juxtacortical lesions, periventricular lesions was noted in 3 cases, spinal cord in 1patient, and 1 had lesions in the infratentorial. A single brain region was affected in 3 patients (Table 1).

**Table 1:** Biographic and clinical characteristics of 6 patients with central nervous system demyelinating syndromes with systemic lupus erythematosus before and after belimumab treatment.

Patient	1	2	3	4	5	6
Sex, Age (yr)	Female, 66	Female, 24	Female, 43	Female, 26	Female, 44	Female, 54
Initial onset of CNS-DS	Yes	No	No	No	Yes	Yes
Duration of disease	6 mo	1 yr	1 mo	5 yr	5 mo	1 mo
Clinical manifestations	Arthritis, Skin rash, Alopecia, Panhematopenia, Lymphocytopenia	Fever, Skin rash, Oral ulcer, Leukopenia	Arthritis, proteinuria	Skin rash, Alopecia, Arthritis, Pleurisy, Leukopenia	Arthritis, Fever, Oral ulcer, Alopecia, Leukopenia	Fever, Muscular pain, Arthritis, Fatigue,
Clinical indexes	Leukopenia, Low Hb, High ESR, C3/C4↓, Anti-sm+, Anti-ANA+, Anti-rRNP+	Leukopenia, High ESR, C3/C4↓, Anti-dsDNA+, Anti-ANA+, Anti-sm+,	Leukopenia, High ESR, C3/C4↓, Anti-dsDNA+, Anti-ANA+, Anti-sm+, Anti-Ro-52+	Low Hb, High ESR, C3/C4↓, Anti-ANA+, Anti-rRNP+, Anti-Ro-52+, Anti-SSA+,	Leukopenia, High ESR, C3/C4↓, Anti-ANA+, Anti-rRNP+, Anti-sm+, Anti-SSA+,	Leukopenia, Low Hb, High ESR, C3/C4↓, Anti-dsDNA+, Anti-ANA+, Anti-SSA+, Anti-SSB+
CNS manifestations	Headache Mood disorder, involuntary mandibular movements	Sensory deficits	Motor deficits	Sensory deficits, Motor deficits	writing disorder, non-fluent aphasia	Sensory deficits, Motor deficits
MRI lesions	Demyelination on Periventricular and juxtacortical lesions; multiple cerebral artery stenosis	Juxtacortical lesions	Juxtacortical lesions	Spinal cord	Periventricular and juxtacortical lesions	Periventricular, juxtacortical, and infratentorial lesions; multiple bilateral microharmorrhagic foci
CNS	Protein and Glucose: Normal	Protein and Glucose: Normal	Protein and Glucose: Normal	Protein increased; Glucose: Normal	Protein and Glucose: Normal	Protein and Glucose: Normal
IgG index/Oligoclonal bands	Normal/None	Normal/None	Normal/None	Normal/Type II	Normal/None	Normal/None
Previous treatment	MP (80 mg, iv), Pred (60 mg, oral Qd), IV-CY,^[10]^ HCQ (200mg), IVIG	MP (60 mg, iv), Pred (60 mg, oral Qd), HCQ (200mg), MTX, IVIG	MP (40 mg, iv), Pred (40 mg, oral Qd), HCQ (200 mg bid), MMF (0.75 g bid), IVIG	MP (500 mg iv pulse 3 + 80 mg, iv), Pred (60 mg, oral Qd), HCQ (200 mg bid), IV-CY,^[8]^ IVIG	MP (500 mg iv pulse 3 + 80 mg, iv), Pred (60 mg, oral Qd), HCQ (200 mg bid), IV-CY,^[8]^ PE^[5]^ and IVIG	DEX (10 mg iv), MP (40 mg, iv), Pred (40 mg, oral Qd), HCQ (200 mg bid), IV-CY,^[3]^ PE^[2]^ and IVIG
SLEDAI-2000	28	18	14	21	20	28
CNS manifestations After treatment	Resolution of headache and involuntary mandibular movements	Resolution of sensory deficits	Resolution of motor deficits	Resolution of Motor deficits, partially remission of Sensory deficits	Relapse when an uterine fibroid removal surgery	Improvement of sensory and motor deficits
MRI lesions after treatment	Multiple cerebral artery stenosis disappeared; improvement in MRI with demyelination on periventricular and juxtacortical lesions	Improvement in brain MRI	Improvement in brain MRI	No change of Spinal cord	Increased brain MRI lesions	Improvement in brain MRI
Duration of remission (mo)	5 mo	12 mo	18 mo	6 mo	/	3 mo

CNS-DS = central nervous system demyelinating syndromes, CY = cyclophosphamide, Dex = dexamethasone, ESR = erythrocyto sedimentation rate, For IV-CY = PE and IVIG numbers in parentheses represent the number of treatments, For MP and DEX = Hbhaemoglobin, HCQ = hydroxychloroquine, IVIG = intravenous immunoglobulin, MMF = mycophenolate, MP = methylprednisolone; pred, prednisone, MRI = magnetic resonance imaging, MTX = methotrexate, PE = plasma exchange, SLEDAI = Systemic Lupus Erythaematosus Disease Activity Index, the doses in parentheses represent maximum dosage.

Before treatment with belimumab, the patients were treated with moderate to high doses of corticosteroids (40–60 mg of prednisolone) and immunosuppressants, which were continued but gradually reduced during belimumab treatment. The table provides details of the clinical symptoms and laboratory tests before and 3.5 month after belimumab treatment. For example, all patients showed leukopenia, decreased complement C3 and C4 levels, and an increased ESR before treatment. Notably, a cerebrospinal fluid analysis demonstrated that glucose, cell counts, and IgG index were within the normal range; a culture was negative for bacteria and fungi; increased protein; and positive for type II oligoclonal bands in 1 patient.

After Belimumab treatment, improvement in the skin, joints, and mucocutaneous lesions was rapid. In addition, the fever, fatigue, and other symptoms were alleviated. SLE disease activity was analyzed before and after treatment, which showed a significant decrease in The Systemic Lupus Erythematosus Disease Activity Index (SLEDAI) from 21.5 (range, 28–14) to 5.5 (range, 8–2) (*P* < .001). Moreover, SLEDAI decreased to less than 4 in 4 patients who received belimumab treatment 8 times, and positive anti-dsDNA antibody in 3 patients turned negative after receiving belimumab treatment 5 times. Moreover, neurologic symptoms in 3 of 6 (50%) patients achieved complete resolution with decreased prednisone, 2 patients achieved improvement, and 1 relapsed after uterine fibroid removal surgery during belimumab treatment. We also evaluated the effect of belimumab treatment by comparing MRI results before and after treatment. Four of 6 patients showed improvement of the brain MRI, no change in MRI in 1 case, and only Patient 5 had increased abnormal detection of T2 weighted images of the brain MRI after relapse. Notably, at the time of writing, 5 patients (patients 1, 2, 3, 4, and 6) remained in remission (for 5, 12, 8, 6, and 1 month, respectively). Importantly, in addition to improving SLE activity and clinical symptoms, belimumab also improved the quality of life of patients, and no adverse events occurred during belimumab treatment. Therefore, the overall effect of belimumab is beneficial.

**Figure 1.**
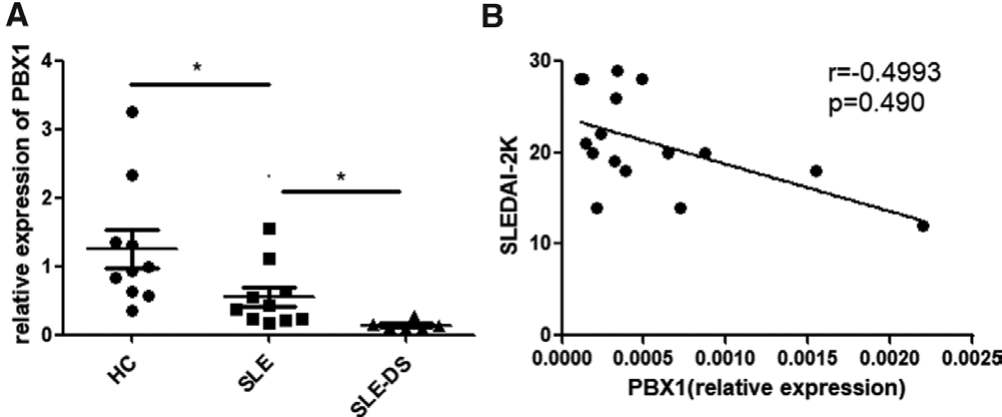
PBX1 expression is downregulated in Peripheral blood B cell of SLE patients, especially in SLE-DS patients, and negatively correlated with SLEDAI score of SLE patients. A. The relative expression of PBX1 in Peripheral blood B cell of SLE, SLE-DS patients and health dornors. B. The correlation between the relative expression of *PBX1* in CD19+ B cells and SLEDAI score from SLE patients. Data are displayed as mean ± s.e.m. *P < .05; **P < .01; ***P < .001 (two-tailed Student *t* test). SLE = systemic lupus erythematosus, SLEDAI = The Systemic Lupus Erythematosus Disease Activity Index, SLE-DS = demyelinating syndrome in systemic lupus erythematosus.

Except for the clinical manifestations and conventional laboratory tests, 10 age- and sex-matched SLE patients without NPSLE and normal controls were recruited to analyze the expression of PBX1, a lupus susceptibility gene, in peripheral blood B cells (Table 2). We found that PBX1 expression in CD19^+^ B cells was lowest in SLE-DS patients than in SLE patients and healthy donors (Fig. 1A), and the expression of PBX1 in CD19^+^ B cells negatively correlated with SLEDAI in SLE patients (Fig. 1B), which was also proved by the latest research in 2023.^[14]^ Thus, we speculated that PBX1 might serve as a potential biomarker for the clinical diagnosis and treatment of lupus in patients with demyelinating syndrome. More data are needed to verify this point.

**Table 2:** Demographic data.*

Characteristic	Health control†	SLE‡	SLE-DS
Sex (male/female)	0/10	0/10	0/6
Age, yr (mean ± SD)	42.89 ± 15.87	42.33 ± 15.36	42.83 ± 16.13
SLEDAI (mean ± SD)		20.8 ± 5.61	21.5 ± 5.58

SLE = systemic lupus erythematosus, SLEDAI = The Systemic Lupus Erythematosus Disease Activity Index, SLE-DS = demyelinating syndrome in systemic lupus erythematosus.

* SLE and SLE-DS patients were administered standard treatment (glucocorticoids and/or antimalarials and/or immunosuppressants [cyclophosphamide, mycophenolate, methotrexate etc.]).

† Healthy donors had no history of autoimmune disease or any treatment with immunosuppressive agents

‡ Systemic lupus erythematosus (SLE) patients without neuropsychiatric systemic lupus.

## 4. Discussion

According to the 1999 American College of Rheumatology (ACR) nomenclature, CNS demyelinating syndrome is a neuropsychiatric systemic lupus erythematosus (NPSLE) that is almost indistinguishable from MS, a prototypical organ-specific autoimmune demyelinating disease.^[15]^ This differential diagnosis has important practical implications for patient care because apart from glucocorticoids, the drugs used to treat the 2 conditions are different. Moreover, interferon (IFN)-based regimens for MS may carry the potential to trigger flares in patients with lupus, which is a disease with a prominent IFN signature.^[16]^ However, DS in SLE is estimated to be rare (<1%), and few studies have described the clinical characteristics and outcomes of this potentially disabling condition.^[17]^ Thus, the occurrence of DS in patients with SLE presents a diagnostic and therapeutic challenge. In our study, a panel of rheumatologists, neurologists, and experienced radiologists diagnosed SLE-DS in these 6 patients. We found that patients with SLE-DS presented with mild extra-CNS disease mainly manifesting as rash, arthritis, alopecia, leukopenia, and hypocomplementemia, while severe non-CNS manifestations were rare. Notably, 6 SLE-DS patients had a negative IgG index and one had a positive oligoclonal band, which were better differentiated from multiple sclerosis. Our case reports, in concert with available literature,^[18]^ suggest that the majority of SLE-DS patients had juxtacortical lesion involvement and often had lesions only in a single brain territory.

Following an accurate diagnosis, early treatment is of the utmost importance, as there is evidence that this improves long-term prognosis.^[4]^ Furthermore, because delay of the therapy may not be feasible, some authors have proposed treating these patients with SLE if any suspicion of the disease is present, after exclusion of other causes such as infection.^[15]^ Randomized controlled trials and reports on SLE-DS are lacking. We report that all patients responded to belimumab treatment, including their neurological symptoms. In case 5, the patient’s condition improved after belimumab treatment and was subsequently aggravated by uterine surgery. Therefore, Belimumab could be an effective and safe option to treat SLE-DS, even in refractory cases, which requires large-sample clinical data.

To date, very few studies have attempted to describe relatively specific biomarkers for the diagnosis and treatment of SLE-DS. Early research has identified the transcription factor PBX1 as a lupus susceptibility gene located in the classical 1q23 susceptible loci of humans^[19]^ and mouse homologous sle1a sites.^[20]^ Shen et al^[14]^ showed that PBX1 was specifically downregulated in autoimmune B-cells and negatively correlated with disease activity as a therapeutic target in SLE. We found that PBX1 expression, which was negatively correlated with SLE disease activity, was lowest in CD19+ B cells from SLE-DS patients than lupus without demyelination and normal controls. However, the underlying mechanism of PBX1 in the pathogenesis of demyelination in lupus remains unclear and requires further exploration.

## 5. Conclusions

Through case reports, we reported the effect of belimumab on 6 SLE-DS patients, even those who were unresponsive to conventional therapy. Neurological symptoms in 5 patients were relieved or partially relieved, and MRI lesions improved in some patients. The indications for belimumab should be carefully determined. Moreover, we propose that the lupus susceptibility gene PBX1 may serve as a potential biomarker for the clinical diagnosis and treatment of SLE-DS. Further understanding of the pathogenesis of SLE-DS and reports of additional cases are required.

## Acknowledgments

We are particularly grateful to all the people who have provided us with help in our article.

## Author contributions

**Conceptualization:** Ya Liu.

**Data curation:** Ya Liu, Qiuyu Fan, Chao Jia, Qibin Wan.

**Formal analysis:** Ya Liu, Qiuyu Fan, Chao Jia, Qibin Wan, Huiqin Yang.

**Funding acquisition:** Huiqin Yang.

**Investigation:** Ya Liu, Huiqin Yang.

**Methodology:** Ya Liu, Qiuyu Fan, Qibin Wan, Huiqin Yang.

**Project administration:** Ya Liu, Huiqin Yang.

**Resources:** Ya Liu, Qiuyu Fan, Chao Jia, Qibin Wan, Huiqin Yang.

**Software:** Ya Liu, Huiqin Yang.

**Supervision:** Ya Liu, Huiqin Yang.

**Validation:** Ya Liu, Huiqin Yang.

**Visualization:** Ya Liu, Huiqin Yang.

**Writing – original draft:** Ya Liu.

**Writing – review & editing:** Huiqin Yang.
